# Signalling pathways involved in the detection of peptones by murine small intestinal enteroendocrine L-cells

**DOI:** 10.1016/j.peptides.2015.07.019

**Published:** 2016-03

**Authors:** Ramona Pais, Fiona M Gribble, Frank Reimann

**Affiliations:** Wellcome Trust - MRC Institute of Metabolic Science, University of Cambridge, Cambridge CB2 0QQ, UK

**Keywords:** GLP-1, L-cell, Enteroendocrine, Peptones

## Abstract

Glucagon like peptide-1 is an insulinotropic hormone released from intestinal L-cells in response to food ingestion. Here, we investigated mechanisms underlying the sensing of peptones by primary small intestinal L-cells. Meat, casein and vegetable-derived peptones (5 mg/ml), the L-amino acids Phe, Trp, Gln and Ala (20 mM each), and the dipeptide glycine-sarcosine (20 mM) stimulated GLP-1 secretion from primary cultures prepared from the small intestine. Further mechanistic studies were performed with meat peptone, and revealed the elevation of intracellular calcium in L-cells. Inhibition of the calcium sensing receptor (CaSR), transient receptor potential (TRP) channels and Q-type voltage gated calcium channels (VGCC) significantly attenuated peptone-stimulated GLP-1 release and reduced intracellular Ca^2+^ responses. CaSR inhibition also attenuated the GLP-1 secretory response to Gln. Targeting these pathways in L-cells could be used to increase endogenous production of GLP-1 and offer exploitable avenues for the development of therapeutics to treat diabetes and obesity.

## Highlights

• Peptones which are an enzymatic protein hydrolysate elevate intracellular calcium and trigger GLP-1 secretion from primary murine L-cells.
• Our data implicate the calcium sensing receptor CaSR in peptone sensing in small intestinal L-cells.
• Our findings also suggest that transient receptor potential channels TRP and voltage-gated Ca^2+^ channels are also recruited by peptones in the small intestine.
• Targeting these pathways in L-cells could be used to increase endogenous production of GLP-1 and can be exploited as therapeutics in the treatment of obesity and type 2 diabetes.

## 1 Introduction

The gastrointestinal tract forms the largest endocrine organ in the body and in addition to its main roles in food absorption and digestion, it also secretes a wide range of hormones from specialised cells known as enteroendocrine cells (EEC). To date approximately 15 subtypes of EEC have been identified, secreting multiple peptide hormones such as peptideYY (PYY), cholecystokinin (CCK), glucose-dependent insulinotropic polypeptide (GIP) and glucagon-like peptide-1 (GLP-1), which control physiological and homeostatic functions related to metabolism and food intake. GIP and GLP-1 are known as incretin hormones because of their ability to potentiate glucose-dependent insulin secretion [11], [21]. GLP-1 also promotes satiety and weight loss [39], and is used therapeutically for the treatment of type 2 diabetes in the form of GLP-1 mimetics and inhibitors of GLP-1 inactivation by dipeptidyl peptidase-4 [10]. GLP-1 is secreted by enteroendocrine L-cells which increase in number along the length of the gastrointestinal tract.

EEC are found scattered amongst the absorptive and other cell types in the gastrointestinal epithelium and respond directly to the arrival and digestion of nutrients such as carbohydrates, fats and proteins in the lumen, culminating in the secretion of their hormonal products into the circulation. While mechanisms by which L-cells sense carbohydrates have been relatively well characterised, however, pathways underlying protein-induced gut hormone release are still far from fully understood. In the perfused rat intestine, GLP-1 release was triggered by luminal perfusion of a protein hydrolysate known as peptone, and the response magnitude was similar in the upper and lower half of the small intestine [37]. In humans, intraduodenal infusion of glutamine or tryptophan increased GLP-1 release [3]. Similarly oral glutamine triggered GLP-1 secretory responses in human volunteers and patients with type 2 diabetes [15], [35]. Meat hydrolysate has been linked to the activation of MAPKs and stimulation of GLP-1 secretion from STC-1 and NCI-H716 cells [33], and albumin egg hydrolysate increased transcription of the proglucagon gene in the GLP-1 secreting cell line, GLUTag [7].

At the molecular level, meat hydrolysate has been found to recruit the G-protein coupled calcium sensing receptor (CaSR) to cause GLP-1 release from primary colonic L-cells and rodent intestine [9], [25] Activity of the CaSR is known to be modulated by oligopeptides [42] and amino acids, with a selectivity towards L-amino acids, especially the aromatic amino acids, L-phenylalanine and L-tryptophan [5]. Other amino acids, such as glycine and alanine are strong stimulants of GLP-1 secretion from GLUTag cells through activation of the ionotropic glycine receptor [14] but this mechanism was not observed in colonic L-cells in primary culture. Other pathways employed by protein digestion products have been described in colonic L-cells and GLUTag cells, which include: L-glutamine triggered membrane depolarisation via electrogenic Na^+^-dependent transporters followed by opening of L- and Q-type voltage gated calcium channels [38], activation of the orphan G protein coupled receptor GPRC6A by ornithine [28] and electrogenic dipeptide transport via PEPT1 [9].

Another receptor activated by polypeptides, lysophosphatidic acid receptor 5 (LPAR5, GPR92/93) was shown to mediate CCK [4] but not GLP-1 secretion [9]. Amino-acid triggered CCK secretion has also been reported to involve the umami taste receptor dimer Tas1R1/Tas1R3 [8]. The mechanisms involved in the sensing of protein digests by small intestinal L-cells, however, still remain unclear and in this study, we sought to elucidate how peptones trigger GLP-1 secretion from primary small intestinal cultures.

## 2 Methods

### 2.1 Solutions and compounds

All compounds were purchased from SigmaAldrich (Poole, U.K) unless otherwise stated. ω-Conotoxin MVIIC was purchased from Peptide Institute, Inc. (Osaka, Japan), ω-Agatoxin IVA was purchased from Alomone Labs. Calhex 231-HCl was purchased from Tocris Bioscience (Bristol, UK).

### 2.2 Animals

All animal procedures were approved by local ethical review committees and conformed to UK Home Office regulations. GLU-Cre12 mice were created using a bacterial artificial construct as described before [29] and express Cre recombinase under the control of the proglucagon promoter. Labelling of intestinal L-cells with a red fluorescent protein (RFP) was achieved by crossing Rosa26tdRFP reporter mice [24] with GLU-Cre12 mice. To monitor calcium fluctuations in L-cells, these mice were crossed with commercially available ROSA26-GCamp3 reporter mice [44] (Jax stock 14,538) to generate L-cell specific expression of a genetically encoded Ca^2+^ sensor.

### 2.3 Primary small intestinal crypt culture

Small intestinal crypts were isolated and cultured as previously described [32]. Briefly, mice 3–6 months old were sacrificed by cervical dislocation and the duodenum (10 cm distal to the stomach) was excised. Luminal contents were flushed thoroughly with PBS and the outer muscle layer removed. Tissue was minced and digested with Collagenase Type XI and the cell suspension plated onto Matrigel (BD Bioscience, Oxford, UK) pre-coated 24-well plates for GLP-1 secretion experiments or on 35 mm glass bottomed dishes (Mattek Corporation, MA, USA) for calcium imaging.

### 2.4 GLP-1 secretion assay

18–24 h after plating, cells were washed and incubated with test agents made up in 138 saline buffer (in mM; 138 NaCl, 4.5 KCl, 4.2 NaHCO_3_, 1.2 NaH_2_PO_4_, 2.6 CaCl_2_, 1.2 MgCl_2_, and 10HEPES, pH adjusted to 7.4 with NaOH) supplemented with 0.1% BSA for 2 h at 37 °C. For experiments where LaCl_3_ and CoCl_2_ were used, carbonates and phosphates were omitted from the saline buffer and compensated with additional NaCl. At the end of the 2 h incubation, supernatants were collected and centrifuged at 2000 rcf for 5 min and snap frozen on dry ice. Cell were lysed with lysis buffer and centrifuged at 10,000 rcf for 10 min and snap frozen. GLP-1 was measured using a total GLP-1 assay (Meso Scale Discovery, Gaithersburg, MD, USA) and results expressed as a percent of total (secreted + lysate) GLP-1 and normalised to basal secretion in response to saline measured in parallel on the same day.

### 2.5 Calcium imaging

Intracellular calcium concentrations were monitored from small intestinal crypt cultures prepared from GluCre/ROSA26-GCaMP3/ROSA26-tdRFP mice. L-cells were identified by RFP fluorescence and changes in intracellular calcium levels were represented by a change in the intensity of GCaMP3 fluorescence excited at 488 nM. Solutions were perfused continuously at a rate of approximately 1 ml/min. Fluorescence in the presence of the test agent was normalised to the respective mean ratio of the background fluorescence of each cell, measured before the addition and after the washout of the test compound. Imaging was performed using an Olympus IX71 microscope with a 40x oil immersion objective, fitted with a monochromator (Cairn Research, UK) and OrcaER camera (Hamamatsu, Japan). Images were acquired at 1 Hz and analysed, after background subtraction, using MetaFluor software (Molecular Devices, USA).

### 2.6 Microarray analysis

Microarray analysis of total RNA from FACS purified L-cells has been described previously [16], using Affymetrix mouse 430 2.0 expression arrays (Affymetrix UK Ltd., high Wycombe, UK). Expression levels of each probe were determined by robust multichip average (RMA) analysis.

### 2.7 Data analysis

Results are expressed as mean ± SEM. Statistical analysis was performed using GraphPad Prism 5.01 (San Diego, CA, USA). For GLP-1 secretion data, one-way ANOVA with post hoc Dunnett's or Bonferroni tests were performed on log-transformed secretion data, as these data were heteroscedastic. Statistical significance for Ca^2+^ imaging data was assessed by Student’s *t* test. Values were regarded as significant when *p* < 0.05.

## 3 Results

### 3.1 GLP-1 secretion is triggered by peptones and amino acids

**Fig. 1:**
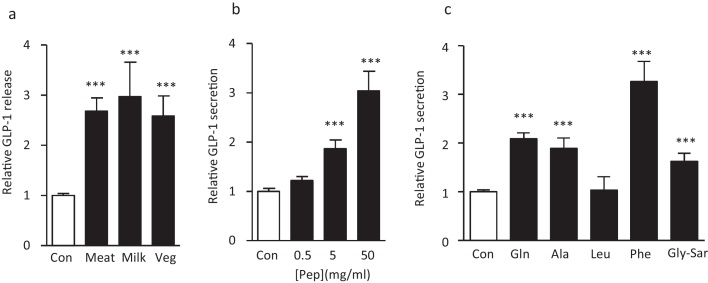
Peptones and amino acids stimulate GLP-1 secretion from small intestinal cultures. (a) Peptones (5 mg/ml) from three different sources stimulated GLP-1 secretion from mixed primary cultures from murine small intestine. Cultures were incubated for 2 h under control conditions or in the presence of meat, milk or vegetable peptones. GLP-1 secretion in each well is expressed relative to the basal secretion (control) measured in parallel on the same day. Data represent the mean ± SEM. ***p  < 0.001 compared with controls by one way ANOVA followed by Dunnet's multiple comparison test. n represents the number of wells, 42 for controls and 31, 12 and 12 for peptones from meat, milk and vegetable source, respectively. (b) Peptones from meat show a dose dependent stimulation of GLP-1 secretion. Cultures were incubated for 2 h under control conditions or in the presence of 0.5, 5 or 50 mg/ml meat peptones. GLP-1 secretion in each well is expressed relative to the basal secretion (control) measured in parallel on the same day. Data represent the mean ± SEM. ***p < 0.001 compared with controls by one way ANOVA followed by Dunnet's multiple comparison test. *n *= 12 wells each for controls and peptones. (c) GLP-1 secretion stimulated by a range of amino acids and the dipeptide Gly-Sar, all tested at 20 mM. Data represent the mean ± SEM. ***p < 0.001 compared with controls by one way ANOVA followed by Dunnet's multiple comparison test. n represents the number of wells for each condition, 36 for controls, 24 for Gln, 15 for Ala, 9 for Leu, 18 for Phe and 12 for Gly-Sar.

Primary crypt cultures were prepared from the top half of the mouse small intestine, cultured overnight and then incubated for 2 h in the presence of test agents for assessment of GLP-1 release. Peptones from three sources, meat, vegetable and casein were tested on the primary intestinal cultures and were similarly effective at stimulating GLP-1 release (Fig. 1a). Meat derived peptones were selected for further experiments and were tested at three different concentrations. Peptones used at 50 mg/ml and 5 mg/ml concentrations significantly stimulated GLP-1 secretion by approximately 3-fold and 2-fold respectively, whereas a lower concentration of 0.5 mg/ml did not elevate GLP-1 release above baseline (Fig. 1b). Additionally, we tested the effects of the L-amino acids glutamine (Gln), alanine (Ala), leucine (Leu), and phenylalanine (Phe) and the non-hydrolysable dipeptide glycine–sarcosine (Gly–Sar), each at a concentration of 20 mM. With the exception of leucine, all these agents significantly elevated GLP-1 secretion (Fig. 1c). The largest response was observed with L-Phe which elevated GLP-1 release 3-fold above baseline (Fig. 1c).

### 3.2 Peptones trigger elevation of intracellular calcium concentrations

**Fig. 2:**
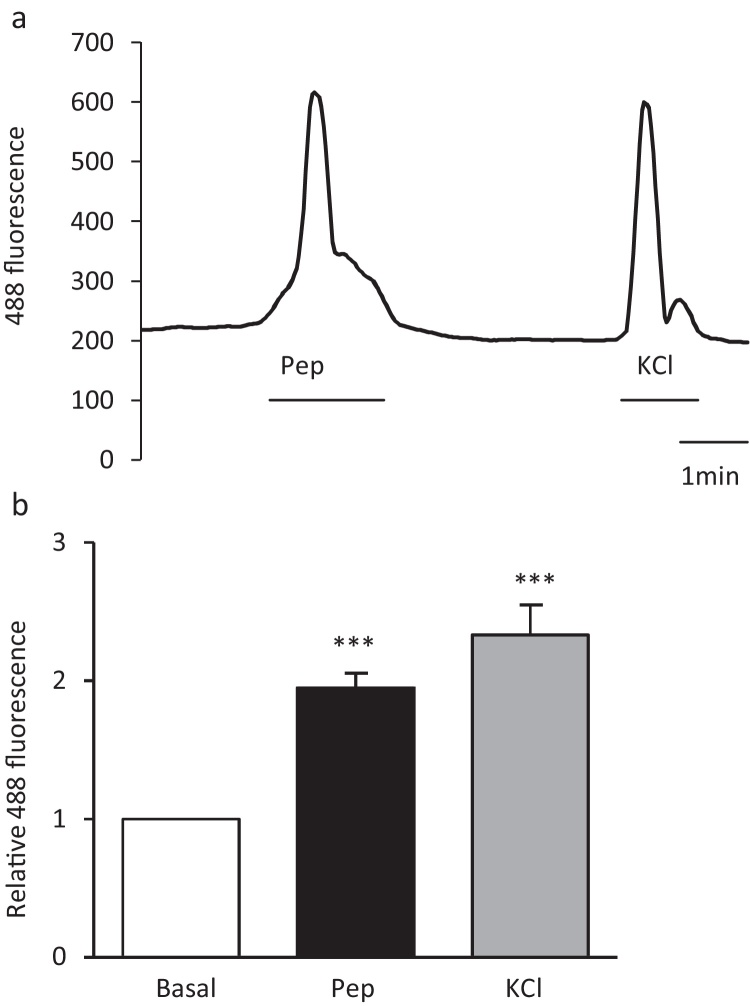
Peptones trigger intracellular calcium concentration elevation in L-cells. (a) A representative trace showing the change in GCaMP3 fluorescence before, during and after the application of meat peptones (pep, 5 mg/ml) to a primary duodenal L-cell cultured from GLU-Cre/ROSA26-GCaMPe mice. Peptones caused reversible increase in cytosolic calcium. KCl (30 mM) was used as a positive control to show that the cell was still viable after peptone application. (b) Mean calcium changes in L-cells following the addition of meat peptone (pep, 5 mg/ml, *n *= 29 cells) and KCl (30 mM, *n *= 24)

In order to elucidate the mechanisms underlying peptone triggered GLP-1 secretion, calcium fluctuations were monitored in primary L-cells cultured from the top half of the small intestine of Glu-Cre/ROSA26-GCaMP3 mice. These mice express the GCaMP3 protein, a genetically encoded Ca^2+^ sensor, specifically in L-cells and hence allow intracellular calcium levels to be monitored by exciting the GFP moiety of GCaMP3. Meat peptones (5 mg/ml) triggered reversible increases in intracellular calcium in L-cells (Fig. 2a and b). We hypothesized that the source of calcium could be release of calcium from intracellular stores or influx of extracellular calcium through either voltage gated calcium channels (VGCC) or non-selective cation channels belonging to the family of transient receptor potential (TRP) channels.

### 3.3 Expression and function of voltage gated calcium channels in L-cells

**Fig. 3:**
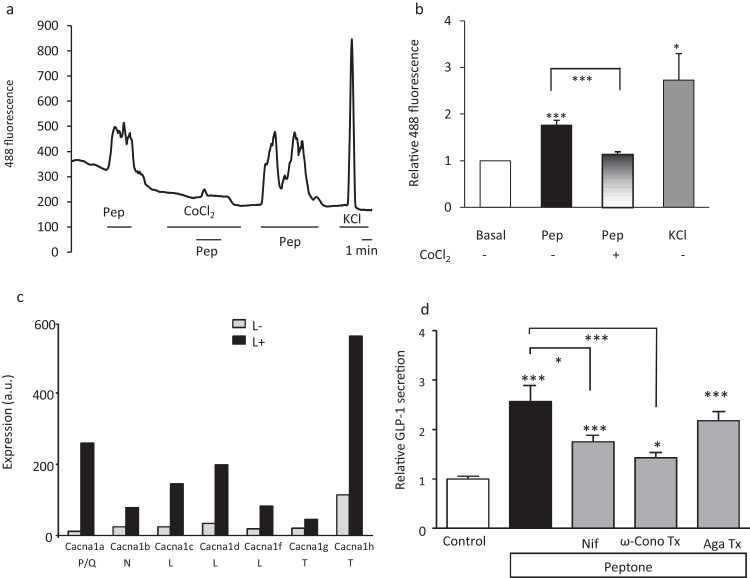
Role of voltage gated calcium channels in peptone-stimulated GLP-1 secretion. (a) A representative trace showing intracellular calcium changes in a primary duodenal L-cell before, during and after the application of meat peptone (pep, 5 mg/ml) and co-application of cobalt chloride (CoCl_2_, 5 mM). (b) Mean calcium changes in L-cells following the addition of peptone in the presence (*n *= 10) and absence (*n *= 10) of cobalt chloride and KCl (*n *= 8). Cobalt chloride significantly inhibited the increase in intracellular calcium by peptones. Data represent the mean ± SEM. *p < 0.05, ***p < 0.001 compared with baseline and between conditions by one- and two-sample Student’s *t* test. (c) Expression of different cacn α-subunit mRNAs in small intestine L-cells (black bars) and control small intestine cells (open bars) assessed by Affymetrix microarray. Expression was evaluated by RMA analysis, and is depicted on an arbitrary scale on which values >100 represent expression that can be reliably detected by quantitative RT-PCR (*n* = 2–3 per cell type). (d) Relative GLP-1 secretion from murine small intestine cultures incubated for 2 h in the presence of meat peptones (*n *= 9) and calcium channel inhibitors, nifedipine (Nif, 10 μM, *n *= 9), ω-conotoxin MVIIC (ω-cono Tx, 1 μM *n *= 9) and ω-agatoxin IVA (Aga Tx, 0.2 μM, *n *= 9). Data represent the mean ± SEM. *p < 0.05, ***p < 0.001 compared with their respective controls by one-way ANOVA with post hoc Bonferroni test.

L-cells are electrically active and respond to nutrient stimuli like glucose, glutamine and di-peptides by membrane depolarisation and activation of voltage gated calcium channels [31] Glutaminereduces postprandial glycemia and augments the glucagon-like peptide-1 response in type 2 diabetes patients. To test the source of the cytoplasmic calcium rise seen in L-cells following peptone treatment, we performed calcium imaging in the presence of cobalt chloride, a general voltage gated calcium channel blocker. Cobalt chloride (5 mM) significantly reduced peptone triggered cytoplasmic calcium rises (Fig. 3a and b), suggesting that peptone stimulation opens voltage gated calcium channels, allowing influx of calcium and GLP-1 secretion.

We compared the expression of the different calcium subunits in L- and non-L-cells from the upper small intestine separated by FACS-sorting and subjected to microarray analysis. As seen in Fig. 3c, L-cells expressed mRNAs encoding P/Q, L, N and T- type calcium channel subunits, all of which were enriched in L-cells over the neighbouring non-L-cells. Colonic L-cells showed a similar pattern of calcium channel subunit expression [34].

Next, we tested the functional relevance of these calcium channels for GLP-1 secretion from primary small intestinal cultures by applying specific pharmacological ion channel blockers [27]. Peptone-stimulated secretion was significantly inhibited by the L-type Ca^2+^ channel blocker nifedipine (10 μM), and the N/P/Q-type Ca^2+^ channel blocker ω-conotoxin MVIIC (1 μM). The P-type Ca^2+^ channel blocker ω-agatoxin (0.2 μM), however, failed to reduce secretion (Fig. 3d).

### 3.4 Transient receptor potential (TRP) channels and their role in GLP-1 secretion

**Fig. 4:**
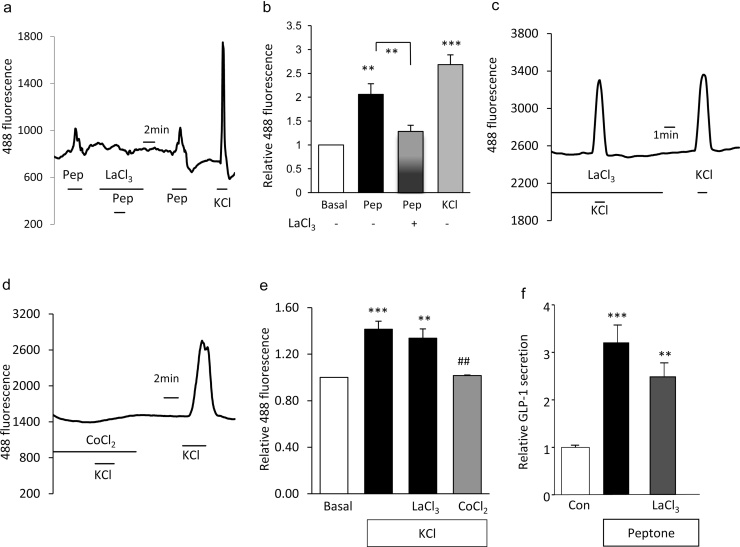
Role of transient receptor potential channels in peptone-stimulated GLP-1 secretion. (a) A representative trace showing intracellular calcium changes in a primary duodenal L-cell before, during and after the application of meat peptones (pep, 5 mg/ml) and co-application of lanthanum chloride (LaCl_3_, 50 μM). (b) Mean calcium changes in L-cells following the addition of peptone in the presence (*n *= 9) and absence (*n *= 9) of lanthanum chloride and KCl (*n *= 7). Co-application of lanthanum chloride significantly inhibited peptone- stimulated rise in intracellular calcium. Data represent the mean ± SEM. **p < 0.01, ***p < 0.001 compared with baseline and between conditions by one- and two-sample Student’s *t* test. (c) Representative trace showing intracellular calcium changes in primary duodenal L-cells before, during and after the application of KCl (30 mM) and co-application of lanthanum chloride (LaCl_3_, 50 μM). (d) Representative trace showing intracellular calcium changes in primary duodenal L-cells before, during and after the application of KCl (30 mM) and co-application of cobalt chloride (CoCl_2_, 5 mM). (e) Mean calcium changes in L-cells following the addition of KCl in the absence (*n *= 17) and presence of lanthanum chloride (*n *= 16) and cobalt chloride (*n *= 6). Co-application of cobalt chloride significantly inhibited KCl- stimulated rise in intracellular calcium. Data represent the mean ± SEM. **p < 0.01, ***p < 0.001 compared with baseline and ^##^*p *< 0.01 compared with KCl alone by one- and two-sample Student’s *t* test. (f) GLP-1 secretion from murine small intestine cultures incubated for 2 h in the presence of peptones (*n *= 19) and TRP channel inhibitors, lanthanum chloride (LaCl_3_, 50 μM, *n *= 19). GLP-1 secretion in each well is expressed relative to the basal secretion (control) measured in parallel on the same. Data represent the mean ± SEM. **p < 0.01, ***p < 0.01 compared with their respective controls by one-way ANOVA with post hoc Bonferroni analysis.

TRP channels form a family of cation conducting channels that respond to a variety of physical, chemical and thermal stimuli [40]. We recently showed by microarray analysis and qRT-PCR, the selective and high expression of *trpa1*, *trpc1*, *trpc3* and *trpm7* in L-cells of the small intestine [12]. Lanthanum chloride (50 μM), an inhibitor of many TRP channels, inhibited peptone induced elevations in cytoplasmic calcium (Fig. 4a and b), suggesting the recruitment by peptones of L-cell TRP channels. Lanthanum did not reduce KCl triggered calcium rises, indicating that it does not exert its effect by blocking voltage gated calcium channels (Fig. 4c and e). Application of cobalt, by contrast, abolished KCl-triggered intracellular calcium elevations (Fig. 4d and e). In secretion experiments, lanthanum, however failed to significantly lower GLP-1 secretion (Fig. 4f).

### 3.5 Extracellular calcium sensing receptor in GLP-1 secretion

**Fig. 5:**
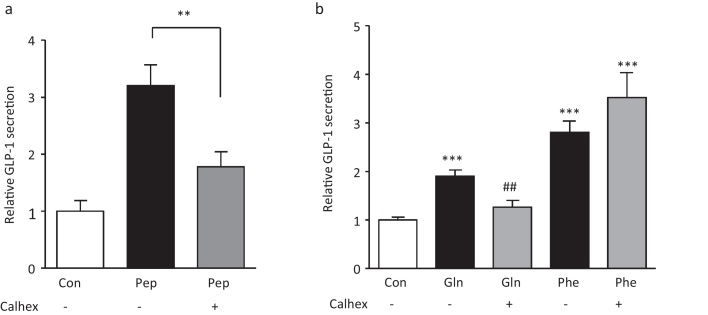
Role of CaSR in peptone-stimulated GLP-1 secretion. (a) CaSR inhibition reduced peptone-stimulated GLP-1 secretion from mixed primary cultures incubated for 2 h under control conditions (standard bath solution, *n* = 19), meat peptone alone (Pep, 5 mg/ml, *n *= 19) or in the additional presence of the CaSR inhibitor, Calhex 231HCl (Calhex, 10 μM, *n* = 16). GLP-1 secretion in each well is expressed relative to the basal secretion (control) measured in parallel on the same. Data represent the mean ± SEM. **p < 0.01, compared with their respective controls by one-way ANOVA with post hoc Bonferroni analysis. (b) Cultures were incubated with Gln (20 mM, *n *= 12) and Phe (20 mM, *n *= 12) in the presence or absence of Calhex 231HCl (10 μM, *n *= 12). GLP-1 secretion in each well is expressed relative to the basal secretion (control) measured in parallel on the same day. Data represent the mean ± SEM. ***p < 0.01, compared with control or ##*p *< 0.01 compared to their respective controls by one-way ANOVA with post hoc Bonferroni analysis.

The CaSR was found to be expressed in the small intestine and colon of mice and highly enriched in L-cells vs. non L-cells [9]. In the presence of Calhex 231 hydrochloride (10 μM) which inhibits the CaSR via negative allosteric modulation [19], peptone stimulated GLP-1 release was significantly reduced 1.8 +/− 0.3 fold stimulation by peptone in the presence of Calhex vs. 3.2 +/− 0.4 fold in its absence (Fig 5a). Calhex also significantly impaired Gln triggered GLP-1 responses, but was surprisingly ineffective at inhibiting Phe triggered secretion (Fig 5b).

## 4 Discussion

Peptones are a commercially available enzymatic protein hydrolysate containing long and short peptide chains and individual amino acids. Meat peptones have reported to stimulate GLP-1 in vitro in human intestinal cell models [33], in perfused rat intestine [37] and following oral administration in rats [17]. In this study, we report that peptones from meat, casein and vegetable sources robustly stimulated GLP-1 release from primary murine small intestine cultures. Further studies performed using meat peptone revealed that this was attributable to the activation of the extracellular calcium sensing receptor, transient receptor potential channels and N/Q and L-type voltage gated calcium channels. GLP-1 secretion from primary L-cells in response to peptones was associated with calcium influx through voltage gated calcium channels, as revealed by calcium imaging experiments performed in the presence of cobalt chloride.

The CaSR senses and responds to extracellular calcium (Ca^2+^_o_) in many tissues and controls calcium homeostasis by regulating parathyroid hormone release from the parathyroid gland [1]. The CaSR also responds to a wide variety of polyvalent cations (Al^3+^, Mg^2+^, and Gd^3+^) other than calcium and even compounds like spermine, polyamines and amino acids. L-Amino acids have been classified as type II calcimimetics, because they act as positive allosteric modulators of the CaSR and require the presence of external calcium to exert their effect. The aromatic L-amino acids Phe, Tyr, His and Trp were found to stereo-selectively enhance the affinity of CaSR for its physiological agonist, Ca^2+^_o_ [6]. In the gastrointestinal tract, CaSR has been gaining reputation as an L-amino acid sensor mediating macronutrient-dependent secretion of gut hormones such as gastrin [13], CCK [23], [43], GIP and GLP-1 [25]. The expression of *casr* is highly enriched in L-cell populations from the small intestine and colon of mice, and its functional relevance in GLP-1 secretion from the colon was shown in primary culture using the agonist calindol, and antagonists NPS2143 and Calhex 231 hydrochloride [9]. Calhex treatment of intestinal I-cell cultures inhibited the effect of Phe and Trp on CCK secretion [43], but rather surprisingly, although Calhex impaired GLP-1 secretion triggered by glutamine and peptones in our study, we were unable to prevent the stimulation of GLP-1 release from small intestinal cultures by Phe. Interestingly, Phe was a highly effective stimulus of GLP-1 release in small intestinal cultures, whereas we reported previously that Phe was not an exceptional stimulus in colonic cultures, and was ineffective in GLUTag cells [32], [38]. The identity of the L-cell sensor underlying Phe- triggered secretion therefore deserves further exploration.

Activation of a number of signalling pathways has been reported following CaSR activation, including the recruitment of G_q_ proteins and intracellular IP_3_ and DAG generation leading to mobilisation of calcium from intracellular stores [6], as well as activation of ERK-dependent signalling pathways [20]. Calcium release from intracellular stores should, however, be insensitive to blockage of voltage gated calcium channels, as we previously reported for the G_q_ coupled bombesin receptor [9]. In this study, we observed a strong inhibition of the peptone-stimulated increase in intracellular calcium by Co^2+^, a non-selective inhibitor of voltage gated calcium channels. We showed previously that cultured primary L-cells are electrically excitable and generate Na^+^ dependent action potentials, in turn activating voltage gated L- and Q-type Ca^2+^ currents, and that GLP-1 secretion in cultures was sensitive to tetrodotoxin under both basal and glutamine stimulated conditions [34]. Similarly, the GLP-1 secreting cell line GLUTag fires Na^+^ channel dependent action potentials [30]. Our findings of cobalt sensitivity of cultured L-cells in calcium imaging experiments indicate the importance of voltage activated calcium currents for calcium responses triggered by peptone. Microarray analysis revealed that this would most likely be attributable to T-, L- and/or P/Q- type channels. Due to the lack of availability of specific pharmacological blockers of the T-type subunit, we were unable to evaluate the functional importance of the low voltage activated T-type channels in mediating peptone responses [18]. Peptone-triggered hormone secretion was, however, lowered by the L-type calcium channel inhibitor nifedipine and the N/P/Q-type inhibitor ω-conotoxin MVIIC, but not by ω-agatoxin, a more specific P-type blocker. Taken together with the expression data, our findings suggest roles for L and Q-type channels, as also reported in colonic L-cells [34].

Although the precise mechanism linking activation of voltage gated channels to CaSR remains unclear, we observed that La^3+^ interfered with peptone stimulated intracellular calcium rises in L-cells. It is thus tempting to speculate that activation of the CaSR could recruit members of the TRP channel. TRP channels act as sensors for osmolarity, mechanical stress, taste and temperature and are divided into seven subfamilies: five group 1 TRPs (TRPC, TRPV, TRPM, TRPN, and TRPA) and two group 2 subfamilies (TRPP and TRPML) [40]. We earlier reported the selective and high expression of *trpa1* on L-cells of the upper small intestine of mice and its activation was shown to cause membrane depolarisation, action potential firing, Ca^2+^ entry and GLP-1 secretion [12]. Other TRP channels such as *trpc1*, *trpc3* and *trpm7* were also enriched in upper small intestine L-cells [12]. Our current data show that lanthanum did not significantly impair peptone-triggered GLP-1 secretion. Lanthanum, although a broad TRP channel blocker, is a strong agonist of the CaSR, activating it at micromolar levels. It enhances the sensitivity of the CaSR to calcium and can even activate it in its absence [2] making it a type I calcimimetic. It is therefore possible that a stimulatory effect of La^3+^ on CaSR over-rode its inhibitory effect on TRP channels in our study.

The sequence of events triggered by peptone and resulting in CaSR activation, TRP channel opening and Ca^2+^ entry through voltage gated Ca^2+^ channels remains to be established. As peptone is a complex mixture of amino acids and oligopeptides, it is possible that distinct components of the mix result in activation of the different signalling pathways. Di/tri peptide transport by PEPT1, for example, was found to trigger voltage-gated Ca^2+^ entry in a previous study [9]. It is also possible, however, that activation of the Gq-coupled signalling pathway downstream of CaSR in some way recruits TRP channels, which in turn result in membrane depolarisation and consequent voltage gated calcium channel opening. Further studies will be required to dissect the cross-talk between these different signalling pathways.

## 5 Conclusion

The enteroendocrine system, through the secretion of a wide spectrum of metabolically active hormones, is profoundly involved in the control of intestinal digestion and motility as well as the stimulation of insulin secretion and regulation of satiety and appetite. Therapeutic strategies to increase the endogenous production of GLP-1 and other gut-derived peptides are currently under development. Our findings reveal the importance of TRP channels and the CaSR in the sensing of oligopeptides by L-cells and could be exploited as a therapeutic target in the treatment of obesity and type 2 diabetes.

## Author contribution

R.P., F.M.G. and F.R. initiated the project and designed the experiments. R.P. performed calcium imaging and GLP-1 secretion experiments. R.P., F.M.G. and F.R. wrote and edited the manuscript.
